# Variables Affecting Medical Faculty Students’ Achievement: A Mersin University Sample

**DOI:** 10.5812/ircmj.14648

**Published:** 2014-03-05

**Authors:** Oya Ogenler, Huseyin Selvi

**Affiliations:** 1 Department of Medical History and Ethics, Mersin University, Mersin, Turkey; 2 Department of Medical Training, Mersin University, Mersin, Turkey

**Keywords:** Education, Education, Medical, Achievement

## Abstract

**Background:**

Training provided in medical faculties is mainly composed of two phases: preclinical and clinical. Preclinical period, or the first three years, consists of theoretical classes and practical implementations to develop vocational skills. In the clinical period, students are given applied courses.

**Objectives:**

This study aimed to determine the role of demographic characteristics and medical students’ life habits on their academic achievement.

**Patients and Methods:**

For this purpose, a 20-item survey form with two sections developed by the researchers was used. Students were also asked to identify the averages of committee exams as the academic achievement indicator. Participating students (n = 287) were from Mersin University Medical Faculty during 2012-2013 session.

**Results:**

Totally, 60.3% of the students were males with an average age of 21.16 ± 1.39, and their general grade point average was 63.39 ± 9.08. Students in their second year (P = 0.000), who were females (P = 0.000), graduated from Anatolian Teachers High Schools (P = 0.002), financially well off (P = 0.026), stayed in state hostels (P = 0.032), did not smoke (P = 0.042) and regularly did sports (P = 0.016) were significantly more successful compared to others.

**Conclusions:**

Students’ socioeconomic resources and habits play roles on academic achievement. Solutions that incorporate economic support which can eliminate negative situations leading to inequality of opportunity among students would increase students' achievement.

## 1. Background

Training provided in medical faculties is mainly composed of two phases: preclinical and clinical. Preclinical period, or the first three years, consists of theoretical classes and practical implementations to develop vocational skills. In the clinical period, students are given applied courses. Physician candidates are required to obtain high academic achievement levels and necessary-sufficient vocational knowledge-skills at the end of the 6-year training course to be graduated (1, 2). Academic achievement levels of physician candidates may be affected by several variables including intelligence, ability, personality, family characteristics, school of graduation, habits, environment, level of welfare, current basic training and the skill of benefiting from provided vocational trainings (1, 3-5). Factors such as loneliness, exam anxiety, current university’s level of the meeting the expectations of students in addition to other segments of society, and the pressure of finding employment after graduation also affect academic achievement (3-5).

Inequality of opportunity problem is observed in cases where the above mentioned variables act as barriers for individuals to obtain educational services (2, 6). In this context, it is vital for physician candidates to access education under equal conditions to provide equal opportunities for everyone, to train high quality physicians and to ensure the human health-safety.

## 2. Objectives

The study had two purposes. The first was to identify the role of demographic characteristics and life habits of medical faculty students on their academic achievement; whereas the second aimed towards establishing and strengthening positive conditions affecting students’ achievement in line with the findings.

## 3. Patients and Methods

This descriptive study was conducted on second-year and third-year students enrolled in Mersin University Medical Faculty between April and May 2013 after obtaining ethical committee and institutional approval (2013/143 of Mersin University Clinical Research Ethics Committee dated 04 November, 2013). Students who did not meet the criteria of studying in the specified university, faculty or semester were not included in the study. Data were collected from 173 of 223 students from the second year, and 114 of 197 students from the third year students who agreed to fill in the survey forms. Target population of the current study included a group of 420 students from second and third years. All students in the university were contacted; however, students who did not agree to participate in the study were excluded since the survey included some personal questions and it was thought that students who were not voluntary would not fill in the survey or answer the questions they were not comfortable with. Therefore, the study was performed with the data collected form 287 students who filled in the survey and answered all the items.

Survey form developed by the researchers was used in the study. The form included 20 items regarding the irreversible and changeable characteristics having a role on student achievement. Student residence, source of income, level of anxiety, study habits, smoking, alcohol intake, sports and artistic habits were considered as changeable characteristics. Gender, age, city of residence before starting university, place of family residence, number of siblings, family income level, parental education level and disease/disability situation were grouped as the irreversible characteristics. Students were given the required information about the study before the implementation of survey and they were allowed not to answer any of the items they did not feel comfortable with. Therefore, students who did not answer a particular question were excluded from the analysis of this item, and each item was analyzed separately. Frequency, means and standard deviation were used in data analysis. Average of committee exams given until the implementation of the form was obtained as the students’ academic achievement level indicator. KR-20 values were calculated to determine the reliability of these exams, and the values obtained between 0.75-0.95 indicated the sufficient reliability. Averages of committee exams and the statistical differences regarding students’ irreversible and changeable characteristics were investigated by using T and F tests. Kolmogorov-Smirnov test was used to check the normal distribution of data (P = 0.200, P > 0.05). In cases where F test provided significant results, Post-Hoc analyses were performed through LSD method and Cohen’s d and eta coefficients were calculated for F and T tests respectively to obtain the strength index for the test. P Value < 0.05 was considered statistically significant.

## 4. Results

Totally, 173 of 223 second-year students and 114 of 197 third-year students participated in the study. Their mean age was 21.16 ± 1.39, and 36.2% (104) of the participating students were females and 63.4% (182) were males, while one participant did not state his or her gender. General grade point average of the students was 63.39 ± 9.08. It was determined that second-year students were more successful compared to the third-year ones (P = 0.000, P < 0.01), and also female students to male students (P = 0.000, P < 0.01), graduates of Anatolian Teachers High School to graduates of other high schools (P = 0.002, P < 0.01), students with higher income to students with lower income (P = 0.024, P < 0.05), students who did not work to cover expenses to students who worked to meet expenses (P = 0.026, P < 0.05), students with no disease or disability to students with a particular disease or disability (P = 0.032, P < 0.05), students who stayed at state hostels or at home with friends to students who stayed alone (P = 0.041, P < 0.05), students who studied in the study room at their hostels to students who studied at the library or at home (P = 0.020, P < 0.05), students who did not smoke to students who smoked (P = 0.000, P < 0.01), and students who regularly did sports to others (P = 0.001, P < 0.05). It was identified that the number of siblings (P = 0.607, P > 0.05), the province where the family resides (P = 0.791, P > 0.05), the place of residence (P = 0.459, P > 0.05), parental education (P = 0.083, P = 0.746), anxiety before-during exams (P = 0.105, P = 0.067, P > 0.05) and changes in nutritional habits during exam times (P = 0.381, P > 0.05) did not play a role on academic achievement (Tables 1 and 2).

**Table 1.** Distribution of Demographic Characteristics (Irreversible Characteristics) Playing Role on Medical Faculty Students’ Academic Achievement

	No. (%)	Exams, Mean ± SD	T	F	P Value	Cohen’s d – eta
**Classes**			4.48		0.000	0.55
Second-year students	173 (60.3)	65.25 ± 9.07				
Third-year students	114 (39.7)	60.56 ± 7.99				
**Gender**			4.30		0.000	0.44
Female	104 (36.2)	66.29 ± 8.32				
Male	182(63.4)	61.78 ± 8.89				
**High School Graduation**				4.48	0.002	0.939
Normal high school (Duz Lise)	23 (8.0)	61.59 ± 9.06			0	
Anatolian high school (Anadolu Lisesi)	136 (47.4)	63.19 ± 9.37				
Science high school (Fen Lisesi)	91 (31.7)	63.74 ± 7.48				
Anatolian teachers high school (Anadolu Ogretmen Lisesi)	27 (9.4)	67.24 ± 9.77				
Others	10 (3.4)	50.05 ± 5.38				
**Number of siblings**				0.50	0.604	
1	19 (7.06)	62.31 ± 10.1				
2	100 (27.9)	64.03 ± 8.42				
≥ 3	150 (55.76)	63.01 ± 9.08				
**Family living province **				0.42	0.791	
Mersin	65 (22.6)	64.04 ± 9.77				
Adana	68 (23.7)	63.48 ± 9.31				
Hatay	19 (6.6)	64.55 ± 10.59				
Osmaniye	6 (2.1)	63.54 ± 10.03				
Other	129 (44.9)	62.39 ± 8.85				
**Location of residence**			-0.742		0.459	
City center	178 (62.0)	63.15 ± 9.32				
county/town/village	80 (27.8)	64.07 ± 8.87				
**Level of father’s education**				0.50	0.736	
Illiterate	14 (4.9)	62.21 ± 8.81				
Primary school	69 (24.0)	62.50 ± 8.91				
High school	64 (22.3)	64.53 ± 9.27				
University	131 (45.6)	63.09 ± 9.48				
Master’s & PhD	9 (3.1)	62.77 ± 7.25				
**Level of mother’s education **				2.08	0.083	
Illiterate	42 (14.6)	61.00 ± 8.50				
Primary school	98 (34.1)	64.46 ± 10.02				
High school	75 (26.1)	64.49 ± 8.24				
University	67 (23.3)	61.43 ± 9.21				
Master’s & PhD	4 (1.4)	63.62 ± 1.36				
**Average monthly income of family**			-2.26		0.024	0.366
0-999 TL	46 (16.0)	60.41 ± 9.25				
1000 TL and higher	229 (79.8)	63.78 ± 9.17				

**Table 2.** Distribution of Changeable Characteristics Playing Role on Medical Faculty

	No. (%)	Exams, Mean ± SD	T	F	P Value	Cohen’s d - eta
**How do you meet your expenses?**			2.24		0.026	0.854
I am working	8 (2.7)	55.64 ± 8.12				
My parents meet my costs/ I have a scholarship	210 (73.2)	63.49 ± 9.17				
**Do you have an illness that could affect your studies?**			2.16		0.032	0.588
Yes	14 (4.9)	58.14 ± 9.27				
No	270 (94.1)	63.54 ± 9.11				
**What is your anxiety level before exams?**						
Low	71 (24.7)	61.88 ± 9.66		2.27	0.105	
Medium	96 (33.4)	64.79 ± 8.69				
High	114 (39.7)	62.87 ± 9.20				
**What is your anxiety level at the time of exams?**				2.72	0.067	
Low	125 (43.6)	64.26 ± 9.29				
Medium	73 (25.4)	63.97 ± 8.01				
High	81 (28.2)	61.35 ± 9.79				
**Where do you live?**				2.35	0.041	0.040
Home alone (1, 2)	34 (11.8)	59.27 ± 9.41				
Home with friends (1)	133 (46.3)	63.17 ± 9.12				
Private student hostel	22 (7.7)	62.18 ± 8.19				
State hostel	38 (13.2)	66.11 ± 9.12				
With my parents	53 (18.5)	64.23 ± 9.30				
With my relatives	5 (1.7)	66.25 ± 4.33				
**Where do you mostly study your lessons?**				2.95	0.020	0.040
Library (1)	42 (14.6)	61.39 ± 7.98				
Home (1)	166 (57.8)	62.97 ± 9.72				
Room at the student hostel	20 (7.0)	64.45 ± 9.67				
Studying room at the student hostel	31 (8.4)	68.01 ± 7.39				
**How do you mostly study your lessons?**			-0.96		0.337	-0.96
Studying alone	259 (90.9)	63.11 ± 9.17				
Studying with group of 2-3 persons	24 (8.1)	64.99 ± 9.04				
**Do you change your diet when you study extensively?**			0.878		0.381	
Yes	151 (52.6)	62.75 ± 9.04				
No	124 (43.2)	63.75 ± 9.27				
**Do you have breakfast every day?**			0.41		0.676	
Yes	140 (48.8)	63.57 ± 9.74				
No	138 (48.1)	63.11 ± 8.59	8			
**Where do you have your breakfast?**			0.78		0.436	
Home	121 (42.2)	63.89 ± 9.80				
Canteen	125 (43.6)	62.97 ± 8.72				
**Do you smoke?**			4.495		0.000	0.557
No	195 (67.9)	64.86 ± 9.09				
Yes	89 (30.3)	59.74 ± 8.46				
**Do you do sports?**				3.11	0.016	0.042
Never	74 (25.8)	62.43 ± 9.11				
Rarely	91 (31.7)	64.98 ± 10.16				
Sometimes	77 (26.8)	60.93 ± 8.09				
Often	31 (10.8)	63.89 ± 8.20				
Regularly	74 (25.8)	62.43 ± 9.11				

## 5. Discussion

The students take approximately 6900-hour classes during the 6-year training in Mersin University Medical Faculty including 1822-hour theoretical classes and 376-hour practical classes during the first three years. The second-year and third-year students in the working group have 541-hour theoretical and 169-hour practical classes and 668-hour theoretical and 61- hour practical classes respectively. Theoretical classes are more concentrated during the first three years and a total of eight theoretical classes such as history of medicine, anatomy, biochemistry, physiology, histology, biophysics, microbiology and behavioral sciences are included in the first and second years' curriculum. Practical classes are also added to the curriculum to develop vocational skills (Training Guide, Medical Faculty, Mersin University, 2012-2013). Third year classes are different from those of the first two years. A total of 20 different theoretical classes including ethics, pharmacology, nuclear medicine, pathology, radiology, biochemistry and microbiology along with others geared towards surgical and internal sciences are included in the third year curriculum. In addition to these classes, practical classes are more concentrated than those of the first and second years classes provided in the curriculum to develop vocational skills (Training Guide, Medical Faculty, Mersin University, 2012-2013). As can be seen from the above explanation, training provided in Medical Faculties is both extended and concentrated compared to the training provided in other faculties. Students need to study more with denser devotion to be more successful (1, 7, 8).

The current study investigated the variables playing role on the academic achievement of medical faculty students, and it was identified that these variables played roles of varying degrees on the achievement of second and third years' students. Especially the association between socioeconomic situation and life habits and achievement was found to be consistent with the results obtained in the literature (7, 9). We found that second year students were significantly more successful compared to the third year ones. It is interesting that other student characteristics do not affect the difference observed between second and third years' students. The training obtained by the end of third year aims that successful students would be able to differentiate the differences in the tissues and organs affected by pathological processes (Training Guide, Medical Faculty, Mersin University, 2012-2013). In addition, maximum amount of medical clinical information is provided in the third year. Additionally, the amount of information provided to students regarding the clinical branches increases day by day. Decreased academic achievement by the third year could be related to the increased amounts of clinical information which is more varied and denser compared to fundamental sciences (1). Current study showed that type of high school the students graduated from had a significant effect on student achievement. Graduates of Anatolian Teachers high school were more successful compared to students graduated from other types of high schools obtained by post-hoc (LSD) analyses. There are many Studies regarding the association between academic achievement and type of high school graduation in the literature (10). These studies also expressed that graduates of Anatolian high schools are more successful compared to graduates of other high schools. In addition, the finding that female students were significantly more successful compared to male students is similar to the findings of studies undertaken on individuals studying undergraduate degrees (6, 11). Students at university are not children nor adults. There are studies indicating that family characteristics, other than parental effort and willingness, affect student achievement (4, 8, 9, 12). Although there are resources emphasizing the role of student environment on their achievement (3, 13, 14). The current study identified that residency province or settlement did not generate meaningful differences on the level of student achievement. This finding is consistent with the research report indicating that there are no regional differences regarding education (6). Families of 98% of the student participants resided in provinces with similar characteristics in the same region (east of the Mediterranean and South East Anatolia). This situation may explain the finding that province or settlement variable did not lead to meaningful differences on achievement (8). Parental level of education did not lead to meaningful differences on academic achievement of students who participated in the study. However, increased achievement with the increase in maternal education draws attention. There are studies in literature showing the fact that parental education levels affect student achievement both in the short and long term (4, 6, 8, 9, 12). The study identified that increase in family or own income resulted in students’ academic achievement and that students who worked to cover for their expenses were significantly less successful compared to other students. The study conducted by Mersin University Guidance Department on Mersin University students found that 47% of students had an income level of less than 125 USD (Dollars) (15). In the current study, 46% of the participating students stated family income of less than 500 USD as well. It is interesting that students with income average level of 60.41 had a passing grade of 60. According to this finding in the study, students with lower income levels had high probability of failing their classes. The students who fail are either declined scholarships or lose extra-family income options. This situation would put the student in a vicious circle for income level (7, 9). Current study found that place of residence had a significant impact on academic achievement. Post-hoc (LSD) analyses showed that students who stayed at state hostels or at homes with their friends were significantly more successful compared to those who lived by themselves. However, it was identified that studying alone or in groups of 2-3 individuals did not lead to meaningful differences on academic achievement (16). It was found that students who lived alone without their families and studied in the library were less successful than those who lived in hostels and studied at study room. This is consistent with other investigations in the literature (9, 16). There is one large library in Mersin University and a study room inside the Medical Faculty. The public library in Mersin is not suitable for study purposes. It is probably due to this issue that library environment is not sufficiently suitable for study when one considers the load of classes included in medical faculty curriculum. The current study showed that changing nutritional habits during concentrated study periods, having regular daily breakfasts, having breakfast at home or at school cafeteria and level of exam anxiety before or during exams did not have any significant effect on academic achievement. There are resources in the literature regarding the fact that exam anxiety decreases achievement due to resulted negative attitudes and behaviors (16, 17). Nine percent of the students participating in the study stated that they had a disability or a disease affecting their studying. It was identified that students with diseases that could interfere with their studies were significantly less successful than others. There are some studies in the literature focusing on the problems of students with disabilities in university environments caused by functions that they cannot fulfill (18). The current study showed that individuals with disability are less successful compared to healthy individuals, which is in line with other studies. Findings point out to inequality in opportunities and the fact that youth with disability need more support. It is also believed that studies in the literature regarding the needs of disabled youth is not sufficient (18). Totally, 30.32% of the participating students stated their smoking. This finding is consistent with the studies performed in Marmara University in 1994 (33%) (15). However, smoking students were found to be higher than those of Cukurova University (22%), Ataturk University (26%), Ondokuz Mayıs University (24%), Gazi University (29%), Dicle University (14%) and Dokuz Eylul University (24%) Medical Faculty students (15). This finding is similar to the findings regarding adolescent smoking rates in Turkey and Iran (19, 20). Students who did not smoke were more successful than those who did. This is consistent with other findings in the literature (19-21). The fact that students training in health issues smoke at a rate consistent with the general public and at a higher ratio than those enrolled in other universities is worth discussing (20, 21). The study showed that students who regularly did sports were significantly more successful than those who sometimes or never did. Many studies are available emphasizing the fact that physical activities positively affect achievement (22). Family characteristics take the lead among the reasons of inequality in achievement obtained during education. Literature shows that there is an association between education and standards of life and that success is affected by income levels (5, 6, 9, 23). Variables playing a role on achievement lead to inequality of opportunity among students (1, 8, 9). Findings in the current study declared that socioeconomic situations of students prevent achievement in classes. Therefore, to ensure equal opportunities, it is imperative to consider the irreversible conditions of students and their environments and to undertake preventive measures on the changeable conditions. Solutions to support students through different financial sources should be especially developed. In addition, identifying barriers in front of students with disability and removing them, helping students gain healthy life habits such as non-smoking and doing sports regularly, and identifying and removing the negative aspects related to study areas would undoubtedly contribute to student achievement significantly.

## References

[1] Wear D (2003). Insurgent multiculturalism: rethinking how and why we teach culture in medical education.. Acad Med..

[2] White KR (1982). The relation between socioeconomic status and academic achievement.. Psychol Bull..

[3] Hill NE, Taylor LC (2004). Parental school involvement and children's academic achievement pragmatics and issues.. Curr Dir Psychol Sci..

[4] Dubow EF, Boxer P, Huesmann LR (2009). Long-term effects of parents’ education on children’s educational and occupational success: Mediation by family interactions, child aggression, and teenage aspirations.. Merrill Palmer Q (Wayne State Univ Press)..

[5] Alivernini F, Lucidi F (2011). Relationship between social context, self-efficacy, motivation, academic achievement, and intention to drop out of high school: A longitudinal study.. J Educ Res..

[6] Batalden P, Leach D, Swing S, Dreyfus H, Dreyfus S (2002). General competencies and accreditation in graduate medical education.. Health Aff..

[7] Aktop A (2010). Socioeconomic status, physical fitness, self-concept, attitude toward physical education, and academic achievement of children.. Percept Mot Skills..

[8] Esen E (2010). Academic Achievement of Turkish and American Students.. Hum Architecture J Soci Self-Knowl..

[9] Najimi A, Sharifirad G, Amini MM, Meftagh SD (2013). Academic failure and students’ viewpoint: The influence of individual, internal and external organizational factors.. J Educ Health Promot..

[10] Kusurkar RA, Croiset G, Galindo-Garre F, Ten Cate O (2013). Motivational profiles of medical students: association with study effort, academic performance and exhaustion.. BMC Med Educ..

[11] Cassady JC, Johnson RE (2002). Cognitive test anxiety and academic performance.. Contemp Educ Psychol..

[12] Mushtaq I, Khan SN (2012). Factors Affecting Students' Academic Performance.. Glob J Bus Manage Res..

[13] Hango D (2007). Parental investment in childhood and educational qualifications: Can greater parental involvement mediate the effects of socioeconomic disadvantage?. Soci Sci Res..

[14] Whaley AL, Noel LT (2013). Academic achievement and behavioral health among Asian American and African American adolescents: testing the model minority and inferior minority assumptions.. Soci Psychol Educ..

[15] Gizir C, Gizir S, Aktaş M, Göçer S, Ömür S, Yüce G (2010.). Mersin Universitesi Ogrenci profili MEÜ PDR Merkezi, Yayın No 1..

[16] Gurpinar E, Alimoglu MK, Mamakli S, Aktekin M (2010). Can learning style predict student satisfaction with different instruction methods and academic achievement in medical education?. Adv Physiol Educ..

[17] Ranasinghe P, Ellawela A, Gunatilake SB (2012). Non-cognitive characteristics predicting academic success among medical students in Sri Lanka.. BMC Med Educ..

[18] Moore-West M, Heath D (1982). The physically handicapped student in medical school: a preliminary study.. J Med Educ..

[19] Nazarzadeh M, Bidel Z, Ayubi E, Bahrami A, Jafari F, Mohammadpoorasl A (2013). Smoking status in Iranian male adolescents: a cross-sectional study and a meta-analysis.. Addict Behav..

[20] Celikel FC, Celikel S, Erkorkmaz U (2009). Smoking determinants in Turkish university students.. Int J Environ Res Public Health..

[21] Kocabas A, Burgut R, Bozdemir N, Akkoclu A, Cildag O, Dagli E (1994). Smoking patterns at different medical schools in Turkey.. Tob Control..

[22] Bradley J, Keane F, Crawford S (2013). School sport and academic achievement.. J Sch Health..

[23] Sirin SR (2005). Socioeconomic status and academic achievement: A meta-analytic review of research.. Rev Educ Res..

